# Aberrant Function of Learning and Cognitive Control Networks Underlie Inefficient Cognitive Flexibility in Anorexia Nervosa: A Cross-Sectional fMRI Study

**DOI:** 10.1371/journal.pone.0124027

**Published:** 2015-05-13

**Authors:** Nick P. Lao-Kaim, Leon Fonville, Vincent P. Giampietro, Steven C. R. Williams, Andrew Simmons, Kate Tchanturia

**Affiliations:** 1 King’s College London, Institute of Psychiatry, Department of Psychological Medicine, London, United Kingdom; 2 King’s College London, Institute of Psychiatry, Department of Neuroimaging, London United Kingdom; 3 NIHR Biomedical Research Centre for Mental Health at South London and Maudsley NHS Foundation Trust and Institute of Psychiatry, King’s College London, London, United Kingdom; University of Western Ontario, CANADA

## Abstract

**Objectives:**

People with Anorexia Nervosa exhibit difficulties flexibly adjusting behaviour in response to environmental changes. This has previously been attributed to problematic behavioural shifting, characterised by a decrease in fronto-striatal activity. Additionally, alterations of instrumental learning, which relies on fronto-striatal networks, may contribute to the observation of inflexible behaviour. The authors sought to investigate the neural correlates of cognitive flexibility and learning in Anorexia Nervosa.

**Method:**

Thirty-two adult females with Anorexia Nervosa and thirty-two age-matched female control participants completed the Wisconsin Card Sorting Task whilst undergoing functional magnetic resonance imaging. Event-related analysis permitted the comparison of cognitive shift trials against those requiring maintenance of rule-sets and allowed assessment of trials representing learning.

**Results:**

Although both groups performed similarly, we found significant interactions in the left middle frontal gyrus, precuneus and superior parietal lobule whereby blood-oxygenated-level dependent response was higher in Anorexia Nervosa patients during shifting but lower when maintaining rule-sets, as compared to healthy controls. During learning, posterior cingulate cortex activity in healthy controls decreased whilst increasing in the Anorexia Nervosa group, whereas the right precuneus exhibited the opposite pattern. Furthermore, learning was associated with lower blood-oxygenated-level dependent response in the caudate body, as compared to healthy controls.

**Conclusions:**

People with Anorexia Nervosa display widespread changes in executive function. Whilst cognitive flexibility appears to be associated with aberrant functioning of the fronto-parietal control network that mediates between internally and externally directed cognition, fronto-striatal alterations, particularly within the caudate body, were associated with instrumental learning. Together, this shows how perseverative tendencies could be a substrate of multiple high-order processes that may contribute to the maintenance of Anorexia Nervosa.

## Introduction

Anorexia nervosa (AN) is a severe mental illness associated with a lifetime prevalence of 0.9% in females and 0.3% in males [1], and carries the highest mortality rate among psychiatric conditions [2]. It has been suggested that persistent dietary restriction, intense fear of gaining weight and disturbances in bodily perception may be caused and maintained by a combination of socio-emotional and cognitive abnormalities [3]. In particular, people with AN demonstrate an inability to advantageously alter their current behaviour in response to changes in the environment [4,5]. This lack of cognitive flexibility remains present after recovery [4,6], is independent of body mass index (BMI: weight/height^2^) and illness duration [5,7] and has been observed in unaffected sisters [5,6,8] as well as in children whose mothers suffer from AN [9]. As such, inefficient cognitive flexibility has been marked as a possible endophenotype of AN that facilitates persistent appetitive control [3] and could contribute to its development [10].

Using a functional magnetic resonance imaging (fMRI) coupled with a target-detection task, Zastrow et al. [11] found decreased activation in AN in the fronto-striatal network when performing behavioural shifts independently of cognitive shift requirement. These findings are corroborated by an activation likelihood estimation meta-analysis of nine voxel-based morphometry studies showing decreased grey matter volume in the right caudate and right lentiform nucleus in currently ill AN [12], and decreased ACC grey matter in recovered participants [13]. In addition, Zastrow et al. demonstrated dominant activation of the right middle frontal gyrus and bilateral temporoparietal junction in AN, regions that form the fronto-parietal control network (FPCN; [14,15]). These observations suggest that inefficient cognitive set-shifting in AN may in fact be attributed to problematic behavioural shifting, characterised by greater cognitive supervisory control and aberrant function of motivation-related circuitry. However, neuropsychological studies report that set-shifting inefficiencies in AN are characterised by an increase in perseverative errors [4,6], whilst no change is observed in the number of loss-of-set/failure-to-maintain-set scores [4]. Since loss-of-sets occur when participants erroneously shift category following positive feedback, avoiding such errors requires efficient behavioural shifting regardless of the ability to shift between abstract rules. The absence of cognitive set-shifting differences by Zastrow et al. could therefore be methodological; the majority of trials categorised as requiring a cognitive shift did not occur immediately following a rule change. Indeed, a recent fMRI study using the Wisconsin Card Sorting Test (WCST) found differential activation during shifting as opposed to maintaining set in the inferior frontal gyrus and bilateral parahippocampal gyrus [16], demonstrating functional alterations associated with cognitive flexibility above that of behavioural flexibility.

The current study aimed to assess cortical function in AN during cognitive flexibility using event-related fMRI. We chose to use the WCST because it permits investigation of separable forms of set-shifting inefficiencies that have previously been linked with distinct neural patterns. For instance, failure to respond appropriately to negative feedback has been associated with decreased regional cerebral bloodflow in the middle frontal gyrus whereas the inability to keep in mind previously unsuccessful strategies was related to decreased regional cerebral bloodflow in the inferior parietal lobule [17]. Such characterisation may therefore help to improve understanding of the pathophysiology associated with inefficient cognitive flexibility in AN.

The WCST also allows measurement of cortical function during instrumental learning and reinforcement, arguably vital processes for efficient flexible behaviour. Indeed, abnormal activity within the dorsal striatum, which plays a major role in forming stimulus-response and action-outcome associations [18], has been observed during cognitive-behavioural flexibility [11,16] as well as during a reward-based guessing game that in healthy individuals relies on ventral striatal function [19]. Furthermore, people with AN exhibit deficits of implicit category learning, which may be the result of disrupted dorsal striatal dopaminergic pathways [20].

We hypothesised that patients with anorexia would 1) perform poorly on the WCST, particularly with regards to perseverative errors, 2) exhibit abnormal fronto-striatal activity during learning and reinforcement and 3) utilise the fronto-parietal control network to a greater degree than healthy controls (HC) during cognitive set-shifting.

## Methods and Materials

### Participants

A total of 32 women with AN (Age range = 18–41; years of illness = 1–25) were recruited from the South London and Maudsley (SLaM) eating disorder service and from a B-EAT community sample (http://www.b-eat.co.uk/) (Inpatient = 10; Outpatient = 9; Day-care = 6; Community = 7). All AN participants had been diagnosed by eating disorder clinicians as fulfilling DSM-IV criteria and included 24 restrictive and 8 binge/purge subtypes. Fifteen were taking ≥1 psychoactive medications during the study (S1 Table). 32 age-matched HC women (Age range = 22–46) with a BMI of >18.5, EDE-Q score of <3 and no personal history of or first degree relative with a psychiatric illness were recruited from the local community. Exclusion criteria for all participants included a history of brain trauma and/or neurological problems (e.g. epilepsy), pregnancy, claustrophobia, inadequate use of the English language, colour blindness, non-corrected visual impairment and metallic implants.

Prior to scanning, all participants were measured to calculate their current BMI. The research version of the structured clinical interview for DSM-IV disorders (SCID-I;[21]) screened for current Axis I psychopathology. IQ was estimated using the Revised National Adult Reading Test (NART-R;[22]). Self-report questionnaires included the 36-item Eating Disorder Examination Questionnaire (EDE-Q;[23]), 14-item Hospital Anxiety and Depression Scale (HADS;[24]) and 12-item Cognitive Flexibility Scale (CFS;[25]).

### Ethics Statement

This study was carried out in accordance with the Declaration of Helsinki under approval of the National Research Ethics Committee, London (Ref 11/LO/0952). Written informed consent was obtained following complete description of the study.

### Wisconsin Card Sorting Test

**Fig 1:**
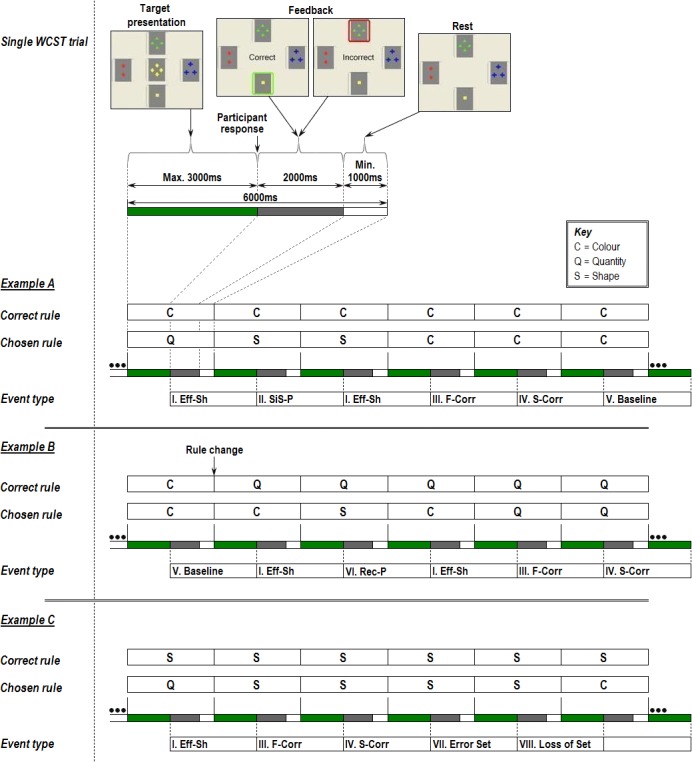
Time course of a WCST trial and events of interest for behavioural and fMRI analysis. Each example presents the current rule chosen by the program (‘correct rule’) with concurrent trial-by-trial participant responses (‘chosen rule’), illustrating different WCST ‘event types’. Note that for event-related fMRI analysis, each event is modelled as the period between two consecutive responses and the ‘event type’ is defined by the combination of the two responses. EXAMPLE A: I) Efficient Shifts (*Eff-Sh*) were when participants changed sorting rules following negative feedback to one that had not been previously tested. II) Stuck-in-set perseverations (*SiS-P*) occurred if the same sorting rule that was incorrect in the previous trial was applied in the subsequent trial. III) First correct sort (*F-Corr*) of a new set. IV) Second correct sort (*S-Corr*) follows first correct sort. V) fMRI baseline (*Baseline*) was designated as trials 3–8 of a string of 8 consecutive correct sorts. EXAMPLE B: VI) Recurrent perseverations (*Rec-P*) constituted shifts to another sorting rule following incorrect feedback, but to one that had been tested two trials previously and already fed back as incorrect. EXAMPLE C: VII) Error Set trials (*Error Set*) were correct sorts not included as baseline trials due to occurrence of ‘loss of set’ errors within that particular set. VIII) Loss of set (*Loss of set*) trials are shifts to a different rule following positive feedback.

Participants performed a modified version of the WCST during the scan. At the start of each trial, a single ‘stimulus’ card was presented in the centre of a grey screen, surrounded by four ‘reference’ cards (Fig 1). ‘References’ were chosen at random at the beginning of each experimental run from a deck of 64 cards that differed along three dimensions; shape (diamond, square, triangle, cross), colour (blue, red, green, yellow) and quantity (one, two, three, four), such that each ‘reference’ was unique on every dimension. ‘Reference’ cards remained unchanged throughout a participants’ experimental run. The remaining 60 cards were used to draw a ‘stimulus’ at random for each trial.

Participants had 3000ms from the start of each trial to match the ‘stimulus’ card to one of the four ‘references’ using a 4-way joystick, according to either shape, colour or quantity. The chosen ‘reference’ was then immediately outlined for 2000ms; in green if correct, in red if incorrect, and the ‘stimulus’ card was replaced with corresponding written feedback. This was followed by a rest period lasting until the trial had elapsed 6000ms, at which point a new trial started (Fig 1). Participants were never directly informed of the correct sorting rule and had to respond based on feedback given at the point of response. The sorting rule at time zero of the experimental run was randomly selected and changed without notice when the participant attained 8 consecutive correct trials (set), in which case the other two dimensions had a 50% chance of becoming the new sorting rule. The task consisted of 100 trials, each lasting 6 seconds, making the total experimental time 10 minutes. Participants were trained prior to scanning to ensure they understood task requirements.

Reaction time and responses were recorded during scanning and classified as discrete events according to previous literature (Fig 1). Notably, we included two forms of perseveration: stuck-in-set (*SiS-P*) and recurrent (*Rec-P*) [17], and adopted the suggestion of distinguishing loss-of-set from efficient shifts (*Eff-Sh*), both traditionally classified as non-perseverative errors [26]. The former is indicative of a disruption in task maintenance. The latter represents adaptive cognitive shifting. First and second correct trials (*F-Corr* and *S-Corr* respectively) were also considered separately for fMRI analysis as they represent learning and reinforcement processes. A high-level baseline of consecutive correct trials was used in order to subtract behavioural flexibility processes from events of interest. Error set, omission, anticipatory and ambiguous trials not conforming to selected event criteria were coded as nuisance covariates.

### fMRI Acquisition

fMRI data were acquired on a 1.5-Tesla GE Signa HDx system running 14m5 software (General Electric Medical Systems, Wisconsin) at the Centre for Neuroimaging Sciences of the Institute of Psychiatry, King’s College London. A body coil was used for RF transmission and an 8-channel head coil for RF reception. T2*-weighted gradient echo echo-planar images (GE-EPI) depicting blood-oxygen-level-dependent (BOLD) contrast were acquired in the axial plane, parallel to the anterior commissure—posterior commissure (AC-PC) line, with the following parameters: repetition time (TR) = 2000ms, echo time (TE) = 40ms, flip angle = 70°, slice thickness = 5mm, slice gap = 0.5mm, field of view = 24x24cm, matrix size = 64x64. Whole-brain coverage was acquired with 25 slices and 300 T2*-weighted whole-brain volumes were acquired for each subject.

To facilitate co-registration of fMRI data in standard space, a whole-brain high-resolution GE-EPI volume consisting of 43 slices parallel to the AC-PC line was acquired for each participant (TR = 3000ms, TE = 40ms, flip angle = 90°, slice thickness = 3mm, slice gap = 0.3mm, FoV = 24x24cm, matrix size = 128x128). Data quality was assured using an automated quality control procedure [27].

### Demographic, Clinical and Performance Analysis

Demographic, clinical and performance data were analysed using Statistical Package for the Social Sciences (SPSS 20.0) [28]. Parametric Student *t*-tests were used to assess between-group differences unless Shapiro-Wilk test indicated non-normality of data, in which case non-parametric Mann-Whitney *U* comparisons were conducted. Performance measures included total number of correct trials, completed sets (8 consecutive correct trials), stuck-in-set perseverations, recurrent perseverations and loss-of-set errors. Reaction times for these events were also analysed, as well as for first correct, second correct and efficient shift trials.

### fMRI Analysis

fMRI data were analysed with XBAM v4.1, developed at King’s College London’s Institute of Psychiatry, which utilises a non-parametric permutation-based approach to minimize assumptions and reduce the effect of outliers (c.f. http://brainmap.co.uk).

### Individual brain activation maps

Data were first processed to minimize motion related artefacts [29]. Following realignment, images were smoothed using an 8.8mm full-width half-maximum Gaussian filter, chosen to improve signal-to-noise ratio over the spatial neighbourhood of each voxel. Responses to each event (‘*Baseline*’, ‘*Eff-Sh*’, ‘*F-Corr*’, ‘*S-Corr*’, ‘*Sis-P*’ and ‘*Rec-P*’) were then detected by time-series analysis using a linear model in which each component of the experimental design was convolved separately with a pair of Poisson kernels (λ = 4 and 8 seconds) to allow variability in the haemodynamic delay. Events were modelled as continuous periods between two sequential participant responses, where the type of event was defined by the combination of these two responses (see Fig 1). The best fit between the weighted sum of these convolutions and the time-series at each voxel was computed using the constrained BOLD effect model [30]. A goodness of fit statistic was then computed as the ratio of the sum of squares of deviations from the mean image intensity resulting from the model (over the whole time-series) to the sum of squares of deviations resulting from the residuals (SSQ ratio).

Following computation of the observed SSQ ratio at each voxel, the data were permuted by the wavelet-based method [31]. Repeated application of this method at each voxel followed by re-computation of the SSQ ratio from the permuted data allows (by combination of results over all intracerebral voxels) the data-driven calculation of the null distribution of SSQ ratios under the assumption of no experimentally determined response. Using this distribution makes it possible to calculate the critical value of SSQ ratio needed to threshold the maps at any desired type I error rate.

#### Group activation maps

The observed and permuted SSQ ratio maps for each individual were transformed into the standard space of Talairach and Tournoux [32] using a two-stage warping procedure [33]. First, the average image intensity map was computed for each individual over the course of the experiment. The transformations required to map this image to the structural scan for each individual and then from structural space to the Talairach template are then calculated by maximising the correlation between the images at each stage. These transformations are then applied to the SSQ ratio maps. Group activation maps were then computed by determining the median SSQ ratio at each voxel (over all individuals) in the observed and permuted data map. Computing intra and inter participant variations in effect separately constitutes a mixed effect approach, which is desirable in fMRI. Detection of activated voxels was extended from voxel to 3D cluster-level using the method described by Bullmore et al. [34]. Resulting cluster-level maps were then thresholded to ensure <1 expected type I error cluster per map. Unlike Bonferroni-based multiple comparison correction procedures, use of the false discovery rate (FDR) ensures adequate control over the type I error rate whilst preventing over-inflation of type II error [35].

#### Group and event comparisons

Between and within-group comparisons were performed by fitting the data at each intracerebral voxel at which all subjects have non-zero data using a linear model of the type:

Y=a+bX+e

where ‘Y’ is the vector of SSQ ratios for each individual, ‘X’ is the contrast matrix for the particular inter-group/inter-event contrasts required, ‘a’ is the mean effect across all individuals in the various groups/events, ‘b’ is the computed group/event difference and ‘e’ is a vector of residual errors. The model is fitted by minimising the sum of absolute deviations rather than the sums of squares to reduce outlier effects. The null distribution of ‘b’ is computed by permuting data between groups or events (assuming the null hypothesis of no effect of group membership or WCST event) and refitting the above model 50 times at each voxel and combining the data over all intracerebral voxels. *F-Corr* vs. *S-Corr* (learning) and *F-Corr* vs. *Eff-Sh* (cognitive flexibility) by group interactions were performed using split-plot analyses of variance (ANOVA) in order to ascertain whether any brain regions exhibit group dependency in differential event-related activation patterns.

## Results

### Demographic, Questionnaire and Performance Measures

The AN group had a significantly lower BMI and cognitive flexibility scale score and higher EDE-Q global, HADS-depression and HADS-anxiety scores compared to HC (Table 1). There was no difference in age but there was a trend for IQ in which the AN group were 6 points lower than HC.

**Table 1:** Demographic and questionnaire measures for Anorexia Nervosa (AN) and Healthy Control (HC) groups.

	Anorexia Nervosa (n = 32)		Healthy Control (n = 32)				
	**Mean**	**S.D.**	**Mean**	**S.D.**	***t* (df)**		**p-value**
**Current BMI**	16.01	1.53	21.93	1.83	*t*(62) = 13.99		<0.001*
**HADS anxiety**	15.16	3.73	4.34	3.08	*t*(62) = 12.64		<0.001*
**CFS total**	43.41	8.69	60.19	6.77	*t*(58.46) = 8.61		<0.001*
	**Median**	**IQR**	**Median**	**IQR**	***U* (df)**	***Z*-score**	**p-value**
**Age**	23	21–30	25	24–28	*U*(1) = 408	1.4	0.161
**IQ (NART)** ^**a**^	110	106–116	116	111–120	*U*(1) = 335.5	2.22	0.027
**EDE-Q Global**	4.05	3.43–4.81	.49	.27-.88	*U*(1) = 3	6.84	<0.001*
**HADS depression**	10.5	7.25–14.75	0.5	0–2	*U*(1) = 23	6.61	<0.001*
**Years of illness** ^**b**^	6.5	3–11.75	-	-			

Score ranges: Hospital Anxiety and Depression Scale (HADS; 0–21), Eating Disorder Examination Questionnaire (EDE-Q; 0–6), Cognitive Flexibility Scale (CFS; 0–72)

(-) indicates where all participants scored 0

IQR = inter-quartile range; S.D. = standard deviation; BMI = body mass index; NART = national adult reading test

(*) indicates results that remain significant after Bonferroni multiple comparison correction

^a^ Data based on 31AN and 32HC

^b^ Data based on 28AN

WCST performance and reaction times are illustrated in Table 2. The AN group were significantly slower than HC when completing loss-of-set trials. No other comparisons remained significant following Bonferroni correction.

**Table 2:** WCST performance and reaction time measures for Anorexia Nervosa (AN) and Healthy Control (HC) groups.

	Anorexia Nervosa (n = 32)		Healthy Control (n = 32)				
**Performance Score**	**Median**	**IQR**	**Median**	**IQR**	***U (df)***	***Z*-Score**	***p*-value**
First Correct	11	10.25–11	11	11–11	*U*(1) = 495	0.26	.796
Second Correct	11	10–11	11	10–11	*U*(1) = 475.5	0.54	.586
Completed Sets	8	7–9	9.5	8–10	*U*(1) = 334.5	2.45	.014
Efficient Shift	14	13–15.75	14	13–15	*U*(1) = 421	1.25	.213
Total Correct	80	75–83	81	79–84	*U*(1) = 399.5	1.52	.129
SiS-Perseveration	.5	0–2.75	1	0–1.75	*U*(1) = 508.5	0.05	.960
Rec-Perseveration	0	0–1.75	0	0–1	*U*(1) = 493.5	0.29	.774
Loss-of-Set	2	1–3	1	1–2.75	*U*(1) = 430	1.12	.261
**Mean Reaction Time (ms)**	**Mean**	**S.D.**	**Mean**	**S.D.**	***t (df)***		***p*-value**
First Correct	1473.9	252.9	1374.3	234.2	*t*(62) = 1.64		.107
Second Correct	1355.1	191.1	1328.6	163.8	*t*(62) = 0.59		.553
Completed Set	1417.3	278.9	1260.4	195.3	*t*(62) = 2.61		.011
Efficient Shift	1426.8	221.2	1288.7	200.0	*t*(62) = 2.62		.011
SiS-Perseveration ^a^	1409.9	562.4	1257.4	314.3	*t*(32) = 0.99		.329
Rec-Perseveration ^b^	1283.4	443.4	1394.4	385.6	*t*(21) = 0.64		.527
Loss-of-Set ^c^	1531.9	401.7	1212.7	269.3	*t*(43.85) = 3.35		.002 *

IQR = inter-quartile range; S.D. = standard deviation

(*) indicates results that remain significant after Bonferroni multiple comparison correction

^a^ Data based on 16AN and 16HC; the remaining participants committed 0 errors

^b^ Data based on 11AN and 13HC; the remaining participants committed 0 errors

^c^ Data based on 26AN and 25HC; the remaining participants committed 0 errors

### fMRI Results: Cognitive Flexibility

Both *F-Corr* and *Eff-Sh* trials are adaptive events that are necessary for the completion of the task. However, these events differ on the basis of the response action carried out; *Eff-*Sh trials are those in which the participant shifts to a different untested rule following negative feedback whilst *F-Corr* trials are those which follow a correct rule application and followed by continued application of that rule. The former requires a shift whilst the latter requires maintenance therefore we reasoned that comparing these events would allow visualisation of neural regions involved in cognitive flexibility.

#### Localisation of regions involved in cognitive flexibility

To identify regions involved in cognitive flexibility, *F-Corr* and *Eff-Sh* trials were compared for each group separately. HC exhibited significantly greater activation in the left precuneus, right anterior cingulate, right middle frontal gyrus, left inferior frontal gyrus and right cerebellum during *Eff-Sh* and in the right putamen, right postcentral gyrus and left paracentral lobule during *F-Corr*. AN exhibited greater activation in the middle frontal gyri bilaterally, left precuneus, right superior frontal gyrus, left medial frontal gyrus, right inferior parietal lobule and left cerebellum during *Eff-Sh* and in the left middle frontal gyrus and left dorsal posterior cingulate cortex during *F-Corr* (Table 3).

**Table 3:** Regions showing a significant difference between Efficient Shift (*Eff-Sh)* and First Correct (*F-Corr*) trials.

			Peak Talairach Coordinates				
Region	BA	Side	x	y	z	Cluster Size (voxels)	*p*-value
**Healthy Control: *Eff-Sh > F-Corr***							
Precuneus	7	L	0	-74	42	152	0.000178
Cerebellum	-	R	47	-59	-29	108	0.000178
Anterior Cingulate Gyrus	32	R	4	19	42	53	0.003018
Inferior Frontal Gyrus	47	L	-47	22	-2	62	0.005859
Middle Frontal Gyrus	9	R	51	15	31	35	0.00799
**Healthy Control: *F-Corr > Eff-Sh***							
Paracentral Lobule	31	L	0	-30	42	127	0.001584
Putamen	-	R	14	7	4	42	0.005631
Postcentral Gyrus	3	R	36	-30	53	45	0.006335
**Anorexia Nervosa: *Eff-Sh > F-Corr***							
Precuneus	7	L	-4	-63	42	302	0.000179
Middle Frontal Gyrus	10	L	-43	44	9	119	0.000359
	9	R	43	30	26	103	0.000359
	6	L	-29	7	53	39	0.003406
Cerebellum	-	L	-33	-56	-24	118	0.000359
Medial Frontal Gyrus	8	L	0	22	42	40	0.003048
Inferior Parietal Lobule	40	R	36	-48	37	55	0.003227
Superior Frontal Gyrus	8	R	36	15	48	31	0.00502
**Anorexia Nervosa: *F-Corr > Eff-Sh***							
Posterior Cingulate Gyrus	31	L	-11	-30	42	781	0.000183
Medial Frontal Gyrus	9	L	-4	37	26	52	0.007156
***Eff-Sh*: AN > HC**							
Precuneus	7	L	-7	-63	48	60	0.000765
Angular Gyrus	39	L	-29	-48	26	27	0.003825

Efficient Shift *(Eff-Sh)*; First Correct *(F-Corr)*; Anorexia Nervosa (AN); Healthy Controls (HC)

All listed regions survived correction for multiple comparisons.

#### Group differences in first correct and efficient shift trials

No differences were found when comparing AN and HC on *F-Corr* trials. There were however differences when comparing groups on *Eff-Sh* trials; AN participants exhibited greater activation in the left angular gyrus and left precuneus (Table 3). There were no areas of greater activation in the HC group.

#### Cognitive flexibility interaction

**Fig 2:**
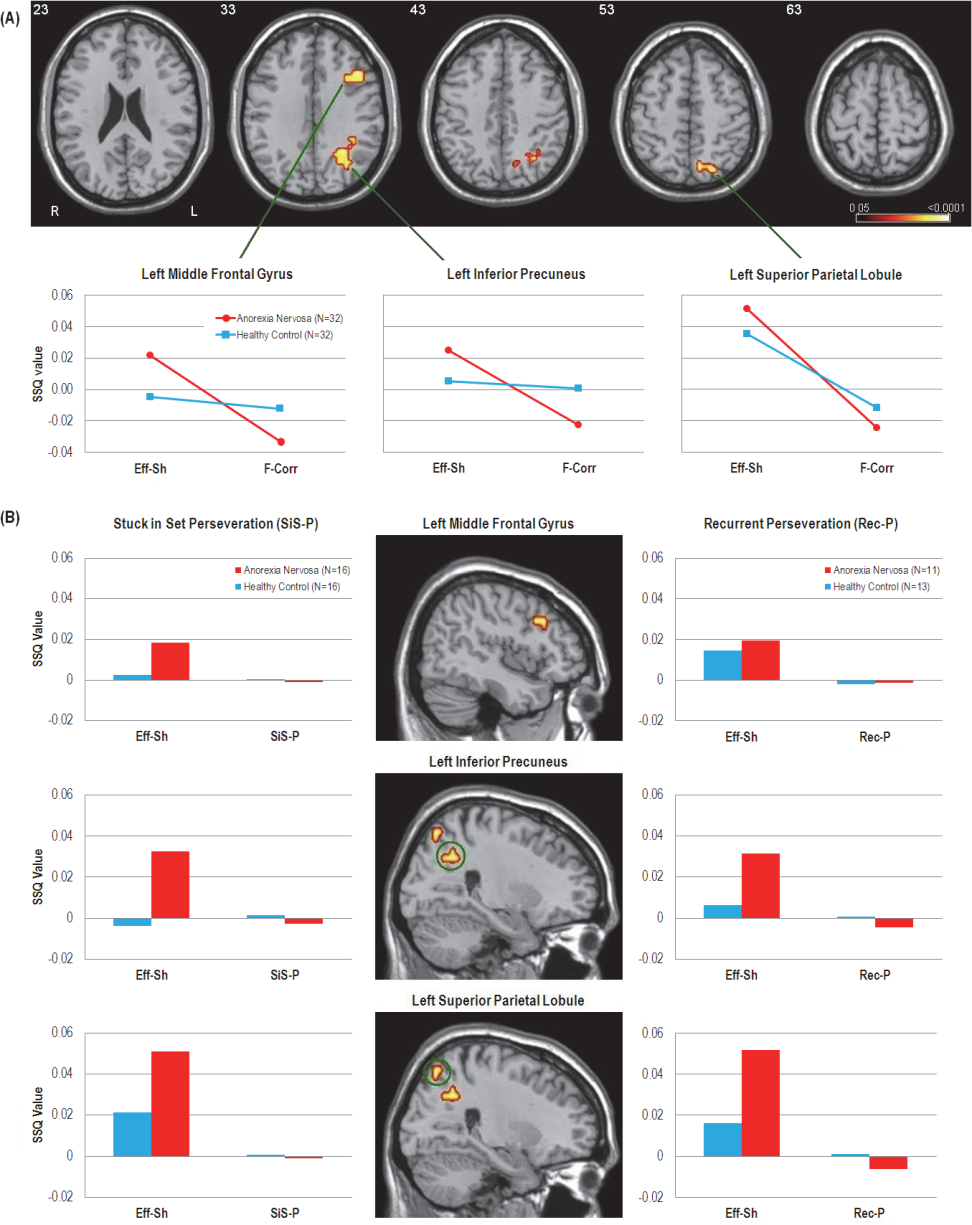
fMRI results of cognitive flexibility comparisons. (A): Axial slices and line graphs showing significant group (Anorexia Nervosa, Healthy Control) x event (Efficient Shift [*Eff-Sh*], First Correct [*F-Corr*]) interactions of BOLD response (SSQ value) in the left middle frontal gyrus (BA9, Peak activation Talairach coordinates = -40, 22, 26, cluster size = 14, p = 0.0069), left inferior precuneus (BA7, Peak activation Talairach coordinates = -25, -56, 26, cluster size = 34, p = 0.0029) and left superior parietal lobule extending into the precuneus (BA7, Peak activation Talairach coordinates = -18, -67, 48, cluster size = 17, p = 0.0078). (B): Sagittal slices and bar charts illustrating the BOLD response during *SiS-P* and *Rec-P* and corresponding activity during *Eff-Sh* in the left middle frontal gyrus, left inferior precuneus and left superior parietal lobule. Functional data were thresholded to yield less than 1 false positive cluster per map and overlaid onto a high resolution single subject T1 structural image in Talairach space.

A group (AN, HC) by event (*F-Corr*, *Eff-Sh*) interaction revealed that the AN group exhibited greater activation during *Eff-Sh* trials but lesser activation during *F-Corr* trials in the left middle frontal gyrus, left inferior precuneus and left superior parietal lobule, as compared to the HC group (Fig 2A).

Only a percentage of participants committed *SiS-P* (50%) and *Rec-P* (37.5%) errors over the course of the task with these participants also showing low event frequency (S2 Table). This meant that there was insufficient power to conduct whole-brain analyses on these event types. Non-parametric voxel statistics (SSQ ratios) for *SiS-P* and *Rec-P* were therefore extracted from significant clusters resulting from the cognitive flexibility interaction analysis and plotted in Fig 2B.

### fMRI Results: Learning

First correct (*F-Corr*) and second correct (*S-Corr*) trials both represent events in which the participant has applied the correct sorting rule. Although a requisite of *F-Corr* is maintenance and application of the rule in the next trial, the period modelled may be associated with uncertainty; first after because the correct rule is found through trial and error and second because the participant may not be sure of the rule even a correct response. As such, the event following repeated application (*S-Corr*) represents verification of what participants believe to be the correct rule. We reasoned that comparing these events would allow assessment of neural mechanisms involved in learning.

#### Group differences in learning trials

**Fig 3:**
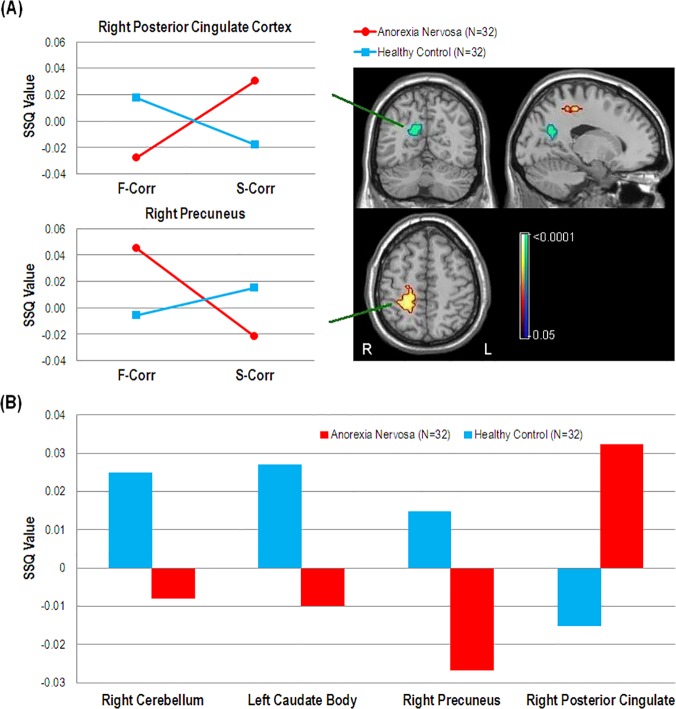
fMRI results of learning comparisons. (A): Coronal, sagittal and axial views of regions showing significant group (Anorexia Nervosa, Healthy Control) by event (First Correct [*F-Corr*], Second Correct [*S-Corr*]) interactions of BOLD response (SSQ value) in the right posterior cingulate cortex (BA31, Peak activation Talairach coordinates = 14, -56, 20, cluster size = 26, p = 0.0071) and right precuneus (BA4, Peak activation Talairach coordinates = 29, -33, 42, cluster size = 33, p = 0.0026). (B): Significant difference in activity between Anorexia Nervosa and Healthy Control groups during Second Correct trials (*S-Corr*) in the right cerebellum (Peak activation Talairach coordinates = 7, -30, -24, cluster size = 36, p = 0.0031), left caudate body (Peak activation Talairach coordinates = -22, -26, 26, cluster size = 30, p = 0.003), right precuneus (BA31, Peak activation Talairach coordinates = 22, -30, 42, cluster size = 21, p = 0.0017) and right posterior cingulate cortex (BA31, Peak activation Talairach coordinates = 11, -56, 20, cluster size = 37, p = 0.0019). Functional data were thresholded to yield less than 1 false positive cluster and overlaid onto a high resolution single subject T1 structural image in Talairach space.

To assess BOLD response during learning, AN and HC groups were compared on *F-Corr* and *S-Corr* trials. As previously stated, there were no differences when comparing groups on *F-Corr* trials. For *S-Corr* however, the HC group exhibited significantly greater activation in the right precuneus, left caudate body and right cerebellum as compared to AN. The AN group exhibited significantly greater activation in the right posterior cingulate cortex as compared to HC (Fig 3B).

#### Learning interaction

A group (AN, HC) by event (*F-Corr*, *S-Corr*) interaction revealed that the AN group had increasing activation in the right posterior cingulate cortex from *F-Corr* to *S-Corr* whereas the HC group exhibited a decreasing trend. Oppositely, AN show decreasing activation in the right precuneus from *F-Corr* to *S-Corr* whilst HC exhibited an increasing trend (Fig 3A).

## Discussion

The current study used the Wisconsin Card Sorting Test (WCST) in conjunction with whole-brain event-related fMRI to elucidate the neural underpinnings of instrumental learning and cognitive flexibility in AN. With regards to behavioural measures, whilst AN attained lower scores on the cognitive flexibility scale and a measure of general WCST performance (completed sets), we did not detect differences with regards to perseverations. Previous neuropsychological studies using the WCST consistently identify heightened perseverative tendency in AN [4,6]. However, neuropsychological WCST protocol refrains from providing information about the rules embedded within the task whilst training sessions are needed to ensure adequate performance in fMRI settings. Indeed, a similar fMRI experiment using the WCST also failed to find perseverative error differences [16]; hence prior strategic knowledge could be responsible for the difference in results.

Despite this, lower activation was found in the left caudate body in AN as compared to HC after consolidating a new rule (*S-Corr*), although no difference was found after first correct trials (*F-Corr*). This was somewhat surprising, given that previous fMRI investigations have found hyperactivity during various executive processes, including reward and cognitive flexibility [16,19] as well as when presented with aversive food stimuli [36]. In addition to facilitating planning and decision-making processes, the caudate nucleus is important for instrumental learning, specifically supporting goal-directed action through formation of action-outcome contingencies [18]. To our knowledge, no studies have directly assessed cortical function during learning processes in AN, although one neuropsychological study provided an indication of disrupted striatal dopamine pathways by showing poorer performance on an implicit category learning task [20]. Since both groups committed an equal number of loss-of-set errors, it is unlikely that decreased caudate activation affected adequate formation of action-outcome contingencies. However, one study of the marmoset medial striatum, an area analogous to the caudate nucleus, showed that focal lesions to this structure cause impairments in reversal learning when goal values and/or task requirements change, whilst the ability to establish new action-outcome contingencies remains intact [37]. Indeed, our AN group exhibited a trend for slower reaction times for shift trials following negative feedback which cannot be explained by general motor deficits as reaction time differences were not widespread, indicating a difficulty in shifting set when prompted. Thus, it is possible that aberrant caudate activation may affect the ability of AN patients to reverse learned associations, which may subsequently produce perseverative tendencies.

We found that activity in the anterior portion of the right precuneus decreased from the first to second correct trials in AN whereas the opposite pattern occurred in HC. This region exhibits high functional connectivity with the superior parietal cortex, paracentral lobule and motor cortex, constituting a network related to sensorimotor processing [38]. Although the exact function of the right precuneus in sensorimotor functions remains elusive, the superior parietal cortex has been found to respond when detection of stimulus features is necessary to update stimulus-response associations [39,40]. Decreasing activation could therefore indicate that people with AN are quicker to assume stimulus-response contingencies than HC. In addition, a recent fMRI study revealed that activity in the caudate, which receives projections from the superior parietal cortex [41], decreases steadily for successive trials following initial acquisition [42]. Although we cannot comment on the activation trajectory of the caudate from first to second correct trials, the fact that it is lower in the second correct trial for AN lends support to this theory of quicker acquisition. Further work is needed to clarify the relative contributions and mutual dependency of aberrant caudate and precuneus activation to learning in AN.

We found a greater increase in activity in AN in the left precuneus and middle frontal gyrus when shifting but greater decrease when maintaining set, as compared to controls. This is in line with Zastrow et al. [11] who demonstrated dominant activation of the fronto-parietal control network (FPCN) in AN during behavioural shifts**.** We add here that differential FPCN activation is also evident during cognitive set-shifting and further, that perseverative errors were characterised by a failure to activate these regions. Interestingly, this lack of activation occurred for both stuck-in-set and recurrent perseverations, suggesting that it is independent of cognitive set-shifting as well as feedback, although due to lack of power this finding should be treated as preliminary. We also found a similar activation difference in the superior parietal lobule extending into the precuneus. This area is part of the dorsal attention network (DAN; [43]), which as aforementioned is linked with perceptual shifting [39,40]. The FPCN shows a high degree of between-network interconnectivity with the DAN and default mode network (DMN), acting as an intermediary that delegates cortical resources towards either externally or internally focussed goal-directed cognition respectively [15,43]. Taken together, these findings indicate that cognitive set-shifting places greater functional demands on people with AN and support current opinion of greater supervisory cognitive control when carrying out external goals [11,19].

### Limitations

Our design used a high-level baseline in an attempt to isolate processes occurring in addition to behavioural flexibility. However, Zastrow et al. [11] showed that the BOLD response associated with behavioural flexibility is different between AN and HC, hence our results may be confounded by our baseline. Although we took this into account by restricting the selection of baseline trials to those that occurred within sets completed with 100% accuracy, and primarily using interaction analyses to visualise the baseline, care must be taken when interpreting the results presented here. We also note that the use of *F-Corr* and *S-Corr* trials to represent learning in the context of cognitive flexibility may be overly simplistic and therefore our results may not be wholly generalizable. However, we maintain that our findings indicate the involvement of learning circuitry in the pathophysiology of AN and thus outline the importance of further neuroimaging investigation.

Perseverative errors were infrequent which due to the noise inherent in fMRI data, caused our analysis to lack power. The WCST is often used in behavioural studies to characterise cognitive flexibility in AN with particular regard to perseveration, therefore it is important to elaborate on its neural bases. The WCST was not designed for use in fMRI settings and it is possible that pre-scan practice sessions together with short task duration resulted in low event count and general lack of behavioural differences. Decreasing the number of trials required to complete a set could increase perseverative error scores whilst keeping the risk of fatigue low and using the practice sessions to measure performance may allow a more valid quantification of cognitive flexibility inefficiencies without confounding practice effects. Alternatively, a block-design method such as that employed by Lie et al. [44] could be used to isolate components of the task via subtraction logic, although this may not allow direct assessment of all WCST event types. Lastly, we found that depression and anxiety scores were higher in AN, however, research suggests that up to 94% of patients with an eating disorder also suffer from at least one comorbid mood disorder [45], therefore we opted to analyse a cohort that reflects clinical presentation.

### Conclusions

Poor cognitive flexibility in AN is characterised by aberrant functioning of the fronto-parietal control network which may contribute to a dysregulation in the way resources are directed between external and internal goal-directed cognition. We corroborate previous work showing no fronto-striatal differences during cognitive flexibility over and above that of behavioural flexibility. However, the finding of altered caudate activation during learning could indicate that people with AN are unable to maintain and/or reverse acquired action-outcome associations. Aberrant learning circuitry may therefore contribute to the observed tendency for people with AN to perseverate and as such, merits further investigation.

## Supporting Information

S1 Table Number of Anorexia Nervosa participants taking psychoactive medications at the time of study. (DOCX)

S2 Table Descriptive statistics of *SiS-P*, *Rec-P* and *Eff-Sh* events occurring in Anorexia Nervosa and Healthy Control groups for perseveration analysis. (DOCX)
