# Supplementary material for: Aberrant Function of Learning and Cognitive Control Networks Underlie Inefficient Cognitive Flexibility in Anorexia Nervosa: A Cross-Sectional fMRI Study
Source: PLoS One. 2015 May 13;10(5):e0124027. doi: 10.1371/journal.pone.0124027 (PMC4430209; doi:10.1371/journal.pone.0124027)
Supplement: S1 Table — (DOCX) [file pone.0124027.s001.docx]

Table S1: Number of Anorexia Nervosa participants taking psychoactive medications at the time of study

| **Medication type** | **No. of participants** | **Specific medications** |
| --- | --- | --- |
| **Anti-depressants** | 5 | Fluoxetine (SSRI) |
|  | 4 | Citalopram (SSRI) |
|  | 1 | Escitalopram (SSRI) |
|  | 1 | Sertraline (SSRI) |
|  | 1 | Venlafaxine (SNRI) |
|  | 1 | SSRI not defined |
| **Anti-anxiolytics** | 2 | Pregabalin |
|  | 1 | Propranolol (Beta-blocker) |
|  | 1 | Not defined |
| **Anti-psychotic** | 1 | Quetiapine |
| **Analgesics** | 1 | Co-codamol |
| **Anti-epileptics** | 1 | Lamotrigine |
