# Supplementary material for: Aberrant Function of Learning and Cognitive Control Networks Underlie Inefficient Cognitive Flexibility in Anorexia Nervosa: A Cross-Sectional fMRI Study
Source: PLoS One. 2015 May 13;10(5):e0124027. doi: 10.1371/journal.pone.0124027 (PMC4430209; doi:10.1371/journal.pone.0124027)
Supplement: S2 Table — (DOCX) [file pone.0124027.s002.docx]

Table S2: Descriptive statistics of SiS-P, Rec-P and Eff-Sh events occurring in Anorexia Nervosa and Healthy Control groups for perseveration analysis

|  | **Anorexia Nervosa** | | | **Healthy Control** | | |
| --- | --- | --- | --- | --- | --- | --- |
| **Stuck in set perseveration groups** | **Median** | **IQR** | **n** | **Median** | **IQR** | **n** |
| Stuck in set perseveration | 2.5 | 1-5 | 16 | 1 | 1-3.25 | 16 |
| Efficient shift | 14 | 13.25-15 | 16 | 14 | 12.75-15 | 16 |
| **Recurrent perseveration groups** | **Median** | **IQR** | **n** | **Median** | **IQR** | **n** |
| Recurrent perseveration | 2 | 1-2 | 11 | 1 | 1-2.5 | 13 |
| Efficient shift | 15 | 13-17 | 11 | 15 | 13-15 | 13 |
